# Emerging optoelectronic architectures in carbon nanotube photodetector technologies

**DOI:** 10.1016/j.fmre.2023.11.001

**Published:** 2023-11-21

**Authors:** Xiaolu Xia, Shaoyuan Zhou, Ying Wang, Zhiyong Zhang

**Affiliations:** a Key Laboratory of Luminescence & Optical Information, Ministry of Education, School of Physical Science and Engineering, Beijing Jiaotong University, Beijing 100044, China; b Key Laboratory for the Physics and Chemistry of Nanodevices and Center for Carbon-Based Electronics, School of Electronics, Peking University, Beijing 100871, China

**Keywords:** Carbon nanotubes, Infrared photodetectors, Device architecture, Diode, Phototransistor

## Abstract

Photodetectors are the fundamental building blocks for many optoelectronic systems, including night vision, optical communications, biomedical imaging, security and motion detection. Carbon nanotubes (CNTs), which have a direct-bandgap structure, a broad spectral response and a large absorption coefficient, provide an ideal research platform for the exploration of high-performance infrared photodetectors. In the past twenty years, great efforts have been devoted to improve detection sensitivity via adopting high-purity CNT films, various doping strategies, optical manipulations and sensitizing nanostructures. Despite considerable strides made, challenges remain in simultaneously achieving high responsivity, low dark current and fast response. In this Review, we summarize recent advances on key device construction strategies and underlying concepts that contribute to improve performance of fabricated CNT photodetectors. The newly emerging heterojunction gated CNT transistors and their potential are highlighted to overcome trade-offs between the optical and electronic processes. Novel applications of CNT photodetectors are further summarized for advanced optoelectronic technologies.

## Graphical abstract

**Figure:**
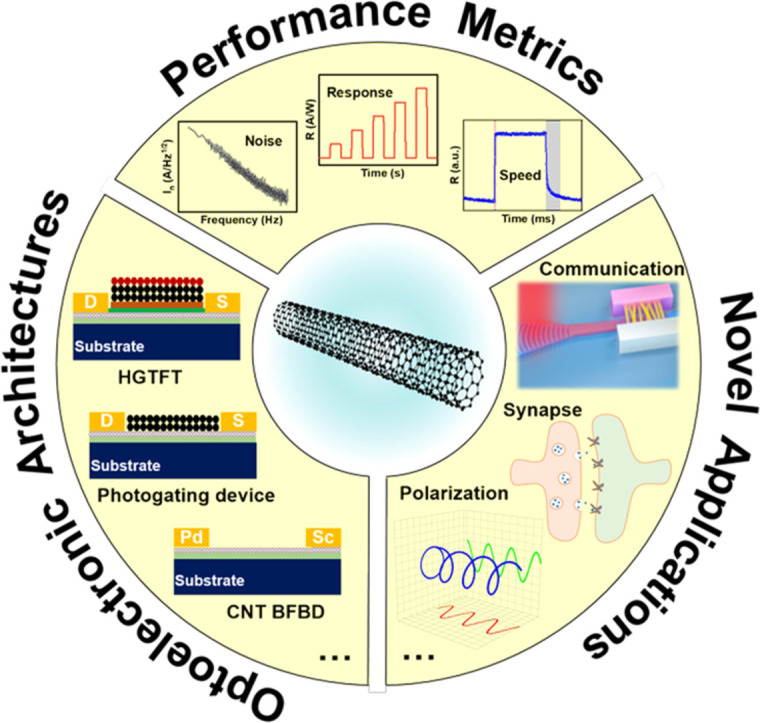

## 1 Introduction

The conversion of light into electrical signals relies on photodetectors, which are revolutionary innovations based on the interaction between light and semiconducting materials. These devices have laid the foundation of many optoelectronic systems that benefit our daily lives, including X-ray imaging, the ubiquitous complementary metal-oxide semiconductor (CMOS) cameras, fiber-optic communication, machine vision and spectroscopy among many others. Historically, most high-end photodetectors are based on highly crystalline semiconductors such as silicon, HgCdTe or III-V compounds, assembled together with modern CMOS readout integrated circuits (ROIC). Photovoltaic-based devices are highlighted for the collaborative optimization of both optical and electronic properties in these bulk semiconductors [1]. After a half-century development, these conventional technologies have reached a high level of maturity and dominated the markets of both consumer electronics and military applications. However, infrared photodetectors based on non-silicon semiconductors always have challenges of high preparation cost, crystal lattice mismatching, low temperature operating requirements, complicated flip-chip bonding process and further scaling down to achieve high resolution [2]. As a result, active research field on novel photodetection grows and different nanomaterials have been employed for light sensing including organic molecules, perovskites, quantum dots, carbon nanotubes (CNTs), graphene and other two-dimensional (2D) materials.

**Fig. 1:**
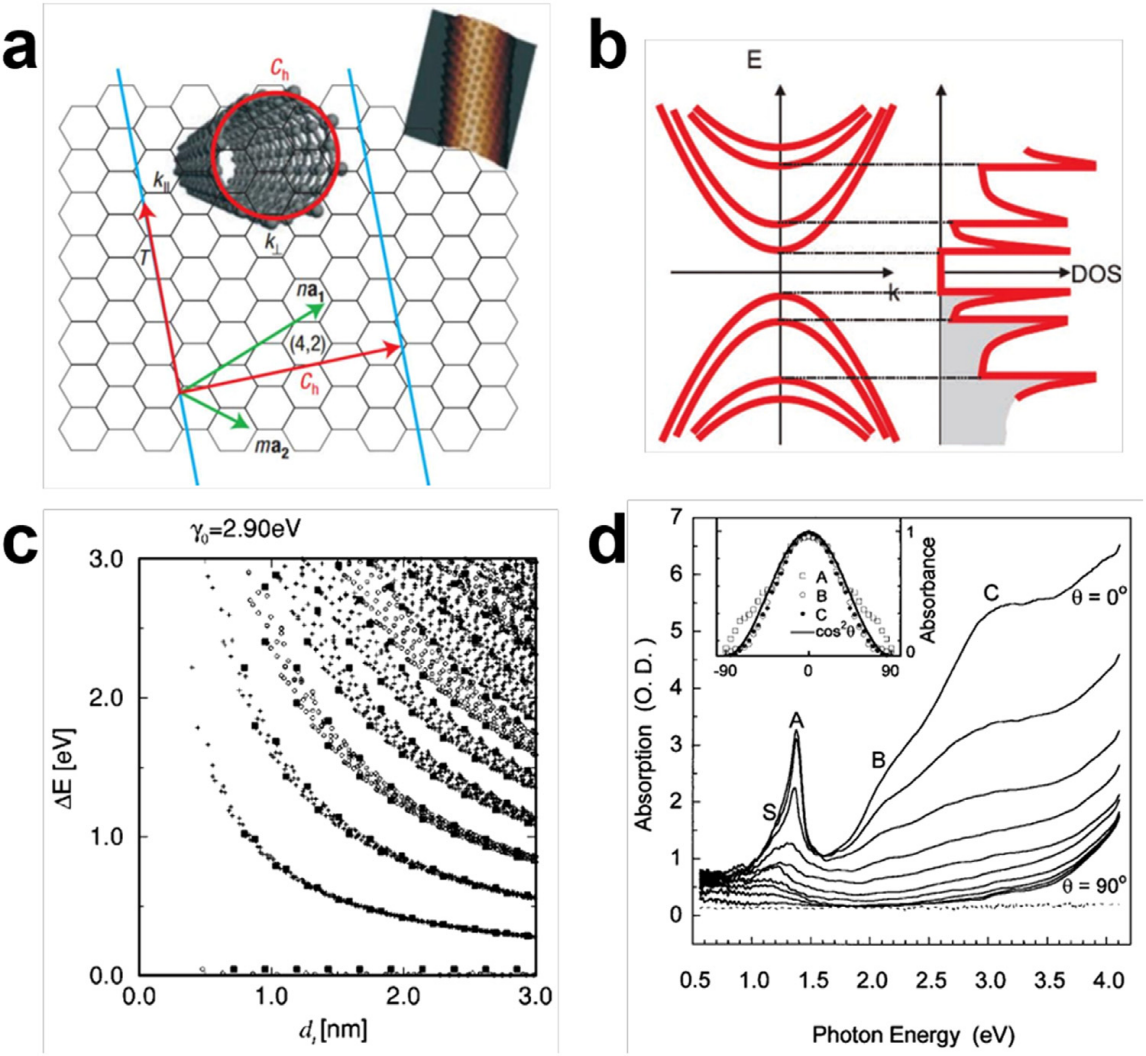
**The geometric, band structure and optical properties of carbon nanotubes.** (a) Structure and vectors for defining a (4,2) CNT. Reproduced with permission from Ref [3], © *Springer Nature* 2007. (b) Schematic electronic energy-dispersion relations and densities of states of semiconducting single-wall CNTs. Reproduced with permission from Ref [4], © *Wiley-VCH Verlag* 2012. (c) Calculation of the energy separations ΔE for all (n,m) values as a function of nanotube diameter (0.7 < *d_t_* < 3.0 nm). The results for different CNTs are based on the tight binding model with *γ*_0_=2.9 eV and *s* = 0 (crosses-semiconducting nanotubes, open circles-metallic nanotubes, filled squares-zigzag nanotubes) Reproduced with permission from Ref [5], © *American Physical Society* 2000. (d) The polarized optical absorption spectra of the single-wall CNTs. Reproduced with permission from Ref [6], © *American Physical Society* 2001.

Single-wall carbon nanotubes (SWCNTs) are regarded as one of the leading candidates to go beyond conventional semiconductors in future electronic circuits and optoelectronics. With the C—C covalent bonds and one-dimensional (1D) geometric structure, SWCNTs exhibit unique physical, chemical and mechanical properties [3]. As shown in Fig. 1a, a SWCNT is composed of hexagonal honeycomb sp^2^ lattice and specified chirality CNTs can be formed with different curled directions (chiral vector), giving rise to distinct properties of either semiconducting or metallic [3]. Based on single-particle model, the fundamental excitation process in CNTs is interband transition that produce free electron-hole pairs. Schematic of the dispersion relation and corresponding densities of states of a semiconducting SWCNT (Fig. 1b) indicate a direct bandgap, which is critical for achieving high absorption coefficient (10^5^ cm^−1^) and quantum efficiency [4]. The bandgap of SWCNT is closely related to its chirality and inversely proportional to its diameter. With the diameters ranging from 0.6 nm to 3 nm, SWCNTs can transduce photons of energies from 0.2 eV to 1.5 eV into electrical signals, covering from visible to mid-infrared (Fig. 1c) [5]. Another advantage of SWCNT-based optoelectronic devices is that the light absorption process is polarization dependent (Fig. 1d) [6]. According to the transition rules, the light absorption along the CNT axial direction is strongest (E_11_ or E_22_ transition), while the absorption for perpendicular polarizations is weak (related to the E_12_ transition). Therefore, filter-free polarization-sensitive photodetectors can be constructed with CNTs.

**Fig. 2:**
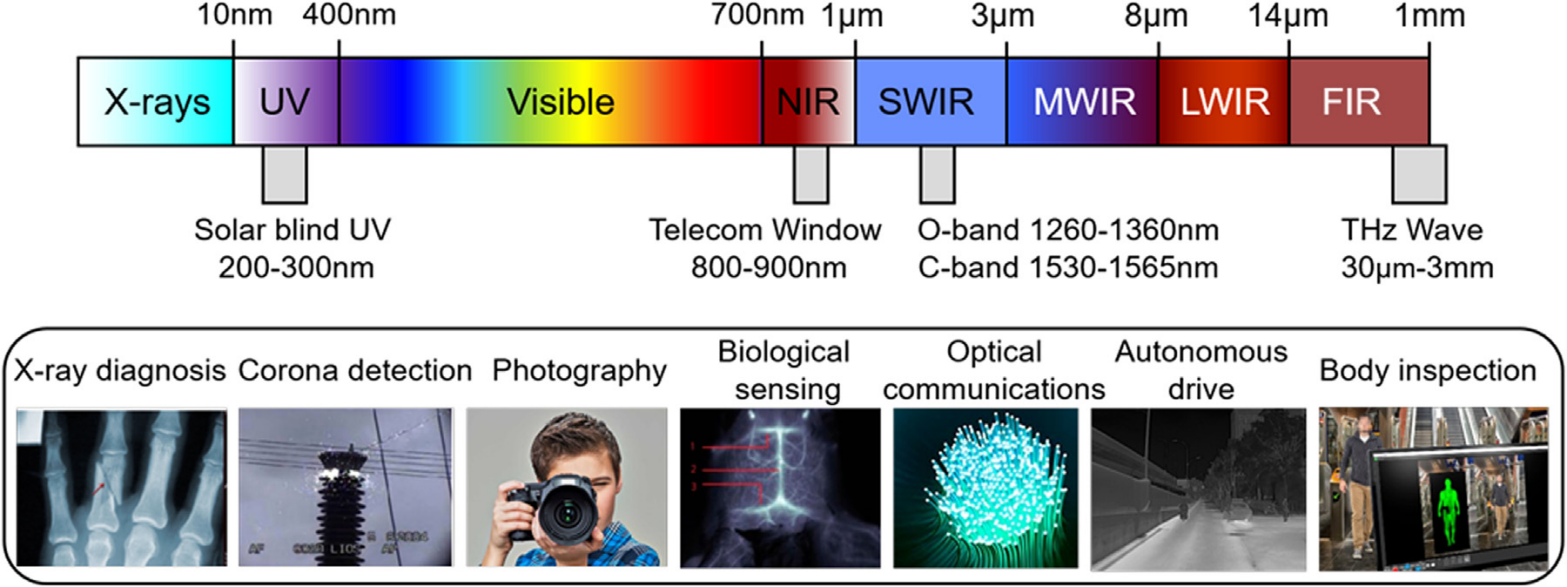
**The wide spectral range afforded by carbon nanotubes allows various applications from the X-ray to the THz spectral region.** UV, ultraviolet; NIR, near-infrared; SWIR, short-wave infrared; MWIR, mid-wave infrared; LWIR, long wave infrared; FIR, far infrared; THz, terahertz.

The development of CNT-based photodetectors goes a long way since the first CNT photoconductor [7]. Numerous materials synthesis methods and device optimization collectively contribute to improve performance of the fabricated photodetectors [8,9]. Typically, high-quality CNT networks and aligned arrays with the semiconducting purity of 99.9999% have been prepared on 4-inch silicon wafers with full wafer coverage [10]. The wide spectral range afforded by CNTs have enabled their prospects in technologies such as X-ray imagers, visible-blind ultraviolet sensor, heart-rate monitors, infrared and Terahertz (THz) detectors, as shown in Fig. 2 [11], [12], [13], [14], [15]. However, CNT devices have rarely exhibited comparable sensitivity to that of commercially available products in spite of their unique advantages. The first challenge is the trade-off between the efficient modulation in a thin channel and the high absorption in a thick channel. In the case of a CNT monolayer, the atomic-thickness channel can be fully depleted by electric field manipulations and operated at extremely low dark currents, while the absorption of photons is rather inefficient and limits the detector response. In the case of a thick CNT film (∼10 µm), CNTs work as a black-body absorber and can absorb more than 99% incident light [16], while the conditions for electronic transport are poor and result in higher dark currents and reduced on/off ratios. The second challenge is the large exciton binding energy, preventing the separation and collection processes of photogenerated electron-hole pairs. Due to the quasi-1D characteristic, free electrons and holes interact strongly, yielding excitons with large binding energies of the order of several hundred meVs [17]. Therefore, by introducing strong built-in electric fields in CNTs can break the exciton binding energy improving the photoelectric conversion efficiency, however, it is difficult to implement by conventional doping technics.

**Fig. 3:**
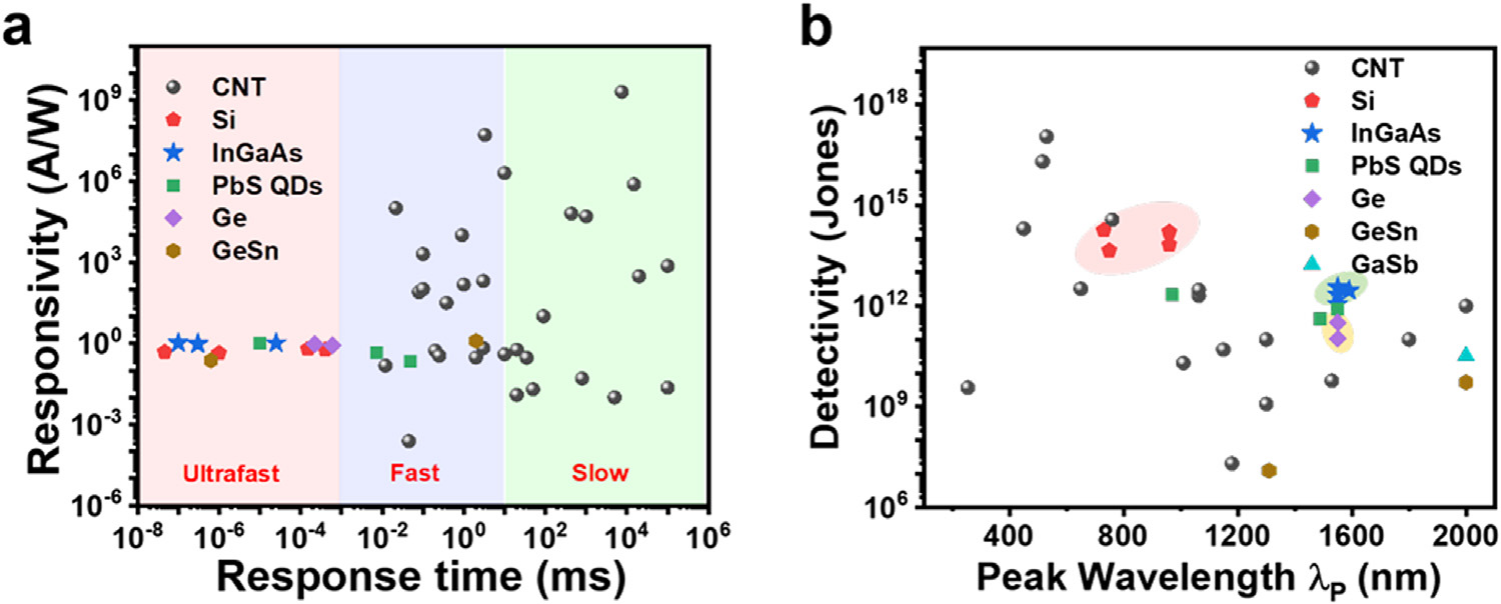
**Comparison of the performances of photodetectors based on CNTs and traditional crystalline systems such as silicon and InGaAs.** The performance data of CNT devices are obtained from Ref [12,14,[18], [19], [20], [21], [22], [23], [24], [25], [26], [27], [28], [29], [30], [31], [32], [33], [34], [35], [36], [37], [38], [39], [40], [41], [42], [43], [44], [45], [46], [47], [48], [49], [50], [51], [52], [53], [54], [55], [56]], and the performance data of commercial products are obtained from the website https://www.thorlabschina.cn/ (SiPD: FDS010, FD11A, FD10 × 10 and FDS025; InGaAs PD: FGA01, FGA015, FGA10 and FGA21; Ge PD: FDG03 and Ge-FDG05).

We benchmark the reported CNT photodetectors with high-performance Si and InGaAs photodiodes (Fig. 3), based on important metrics involving photoresponsivity, response time and peak detectivity [12,14,[18], [19], [20], [21], [22], [23], [24], [25], [26], [27], [28], [29], [30], [31], [32], [33], [34], [35], [36], [37], [38], [39], [40], [41], [42], [43], [44], [45], [46], [47], [48], [49], [50], [51], [52], [53], [54], [55], [56]]. Despite the small absorption cross-section of CNT materials, high response is extensively found in CNT devices typically due to the trap- or hybrid-induced photogating mechanism [30,32,40]. However, the fastest response time obtained in most CNT devices is more than 100 µs, much larger than the intrinsic photoresponse time of CNTs (picosecond) and the response time of Si or InGaAs photodetectors (100 ps ∼400 ns) [29,57]. Meanwhile, the peak detectivity of CNT photodetectors is usually lower than 10^11^ cm Hz^−1/2^ W^−1^ in the infrared regimes, challenged with the breakthrough for improving by two orders of magnitude. Some literatures did not experimentally measure the noise power spectra, and the obtained data in Fig. 3b were exaggerated. These problems make CNT-based photodetectors frustrating, limit their commercial deployment, and turn researchers’ attention to newly developed 2D materials. Whereas, we believe that the above challenges in CNTs also exist in photodetectors based on other low-dimensional semiconductors. To improve the overall performance and realize the technological potential, new strategies are necessary to promote the transduction from light to electronic response in such thin films.

In this Review, we focus on the architecture evolution of CNT photon detectors and survey the optimal optoelectronic architecture for high sensitivity and fast response. We first introduce basic performance metrics of photodetectors and discuss well-established methods for accurate characterization. We put emphasis on the structural designs and fundamental operating principles of each class of photodetector, their individual merits, drawbacks and relative trade-offs. Then, we highlight how the heterojunction-gated CNT thin film transistors overcome of the conventional photodetection limits and achieve a specific detectivity of 5.6 × 10^13^ cm Hz^−1/2^ W^−1^ in the shortwave infrared region. The design of complete opto-electronic decoupling allows high absorption in PbS quantum dots (QDs), and efficient electrical modulation and fast charge transport in the thin CNT channel, ideally without mutual interference. Finally, we discuss recent advances on the novel applications of CNT-based photodetectors.

## 2 Performance metrics of photodetectors

The main photoelectric processes of CNT photon detectors include light absorption, excitons generation, electron-hole separation and collection. To evaluate the pixel-level performance of photodetectors, it is important to evaluate basic performance metrics of responsivity, speed, noise, specific detectivity, and other attributes such as size, cost, power dissipation, ease of integration, etc. However, optoelectronic measurement is a complex issue due to the large number of experimental variables involved. Different electrical and radiometric parameters must be precisely and simultaneously controlled. The characteristics of photodetectors based on novel materials and complex structures have been even more complicated because of different mechanisms. Here, we will describe some universal parameters of photodetectors and discuss detailed measuring methods for CNT-based device characterization.

### 2.1 Responsivity, quantum efficiency and photoconductive gain

The simplest performance metric is the responsivity (*R*(*λ*)) defined as the ratio of photocurrent or voltage generated to the incident optical power falling on the detector at a given wavelength *λ*. According to the absorption spectrum of the adopted semiconductor materials, peak responsivity is often measured and given by the expression:

(1) $$R_{i}\left(\lambda \right)=\frac{I{\left(\lambda \right)}_{ph}}{P{\left(\lambda \right)}_{in}}\;\text{or}\;R_{V}\left(\lambda \right)=\frac{V{\left(\lambda \right)}_{ph}}{P{\left(\lambda \right)}_{in}}$$

where *I*(*λ*)_ph_ is the photocurrent in amperes, *V*(*λ*)_ph_ is the photovoltage in volts and *P*(*λ*)_in_ is the incident optical power in watts. The responsivity of a photodetector often depends on incident wavelength, modulation frequency and applied bias.

External quantum efficiency (*EQE*(*λ*)) represents the ratio of the number of excited electron-hole pairs to the number of incident photons on the active area. The *EQE*(*λ*) is a standardized value, generally less than a unity. Whereas, deviations from the above definition are observed in the high energy of photons or in photodetectors with internal gain, where *EQE*(*λ*) can reach values beyond a unity.

Photoconductive gain (*G*(*λ*)) is often related to the photorecycling effect in traditional photoconductors, due to the big difference in mobility of electrons and holes. For example, the separated photoelectron moves faster in many semiconductors and reaches the positively charged electrode quickly, while the remaining hole continuously induce the flow of new electrons onto the absorbed one photon, until either the hole reaches the negative electrode or recombines with one electron in the channel. This type of photoconductive gain is usually observed in infrared detectors operated at low temperature. For novel low-dimensional materials, photoconductive gain can be achieved by localized states or hybrid structures at room temperature, referred to the photogating effect. The separated electrons or holes are trapped, leading to an obvious increase of the excess minority carrier lifetime (*τ*). The optical gain can thus be estimated by the equation *G*(*λ*) = *τ*/*τ*_T_ (*τ*_T_ is the carrier transit time).

For devices operating in photovoltaic mode and in the absence of any secondary photocurrent, the *R*(*λ*), *EQE*(*λ*) and *G*(*λ*) are interrelated as:

(2) $$EQE\left(\lambda \right)=\frac{I{\left(\lambda \right)}_{ph}}{P{\left(\lambda \right)}_{in}}\frac{hc}{q\lambda }∼R\left(\lambda \right)\frac{1.24}{\lambda }\;\left(\lambda \text{in}\;\mu \;m\right);\;\left(R\left(\lambda \right)=\frac{EQE\left(\lambda \right)G\left(\lambda \right)}{1}\frac{q\lambda }{hc}\right)$$

where *h* is Planck's constant, *c* is the speed of light and *q* is the elementary charge. For devices with internal gain, the *R*(*λ*) is generally used to compare the photoresponse of detectors.

**Fig. 4:**
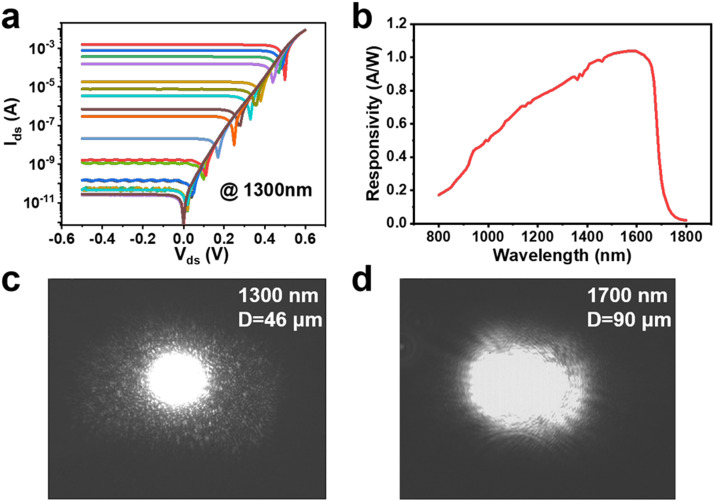
**The determination of incident optical power and intensity distribution.** (a) The photocurrent I_ph_ of a standard InGaAs diode (Thorlabs FGA21) under different light illumination. (b) The calibrated responsivity R_λ_ of the InGaAs diode as a function of wavelength. The incident optical power is calculated according to the formula *P*= I_ph_/R_λ_. The measured beam profile and intensity distribution of 1300 nm (c) and 1700 nm laser spot (d) using the InGaAs camera (First Light Vision C-Red3). The extracted diameters (D) of laser spot are 46 µm and 90 µm, respectively.

Responsivity measurements are essential for characterizing photodetectors. During this procedure, d.c. parametric test with high accuracy can be achieved with precise instruments of semiconductor parameter analyzers and probe stations. The determination of incident light intensity is critical. On one hand, the incident optical power need to be measured by the standard Si or InGaAs photodiode in their linear dynamic range. It shows the photocurrent of an InGaAs diode (Thorlabs FGA21) to the 1300 nm illumination of a super-continuous spectrum laser in Fig. 4a, and it shows the calibrated responsivity (Fig. 4b). Therefore, the incident power at 1300 nm can be calculated by converting a voltage by placing a load resistor ($R_{L}$), following the expression: $V_{O}=P{\left(\lambda \right)}_{in}\times R\left(\lambda \right)\times R_{L}.$ On the other hand, laser beam visualization is necessary for the analysis of beam profile and intensity distribution. For example, it shows that the diameter of laser beam at λ=1300 nm is 46 µm (Fig. 4c), and it shows that the beam profile at λ = 1700 nm slightly deviates from the circle (Fig. 4d). With the measured light power and intensity distribution, we can then calculate the power density and R(λ) of the measured photodetector.

### 2.2 Response time and bandwidth

The temporal response of a photodetector is typically characterized by its time constant (τ) or 3 dB bandwidth (*f*_c_). Time constant (τ) is defined as the time taken by the detector output signal to reach 0.63 of its peak steady-state value in response to an incident optical signal, when the output signal rise or fall according to the exponential relationship. Otherwise, the rise time (*t*_r_) and fall time (*t*_f_) are often used, defined by the photodetector output signal to rise from 10% to 90%, or fall from 90% to 10% of its final value, respectively. The rise time can be related to time constant (τ) as: *t*_r_ = 2.2τ. With increasing modulation frequency *f* of input light pulse, the photoresponsivity ratio *I*(*λ*)_ph_/*I*_d_ decreases because the photocurrent does not decay fully during a modulation period. The responsivity of the modulation frequency *f* follows:

(3) $$R{\left(\lambda \right)}_{f}=\frac{R{\left(\lambda \right)}_{0}}{\sqrt{1+{\left(2\pi f\tau \right)}^{2}}}$$

where $R{\left(\lambda \right)}_{0}$ is the d.c. responsivity of zero modulation frequency. The 3 dB bandwidth of the detector *f*_c_ is defined as the frequency at which the photoresponsivity is reduced from its peak value to 0.707 times, following the expression: *f*_c_ = 1/(2πτ).

The response time of photodetectors generally depends on the lifetime of photocarriers, carrier mobility and external electric field. The rise and fall times can be measured using rectangular optical pulses generated by pulsed laser or pulse-modulated light-emitting diode (LED). The output signal from the photodetector is measured at load resistance with a broadband oscilloscope. In some cases, photodetection speed was determined by measuring the transient photocurrent and reading the time for the photocurrent to decrease from the peak to 1/*e* after a single exponential fit for the photoresponse curve. Although ultrafast response of sub-nanoseconds was obtained, it is not the strict response time for CNT photodetectors. The standard square wave test is more appropriate to confirm the steady-state response of saturation photocurrent.

### 2.3 Electronic noise

**Fig. 5:**
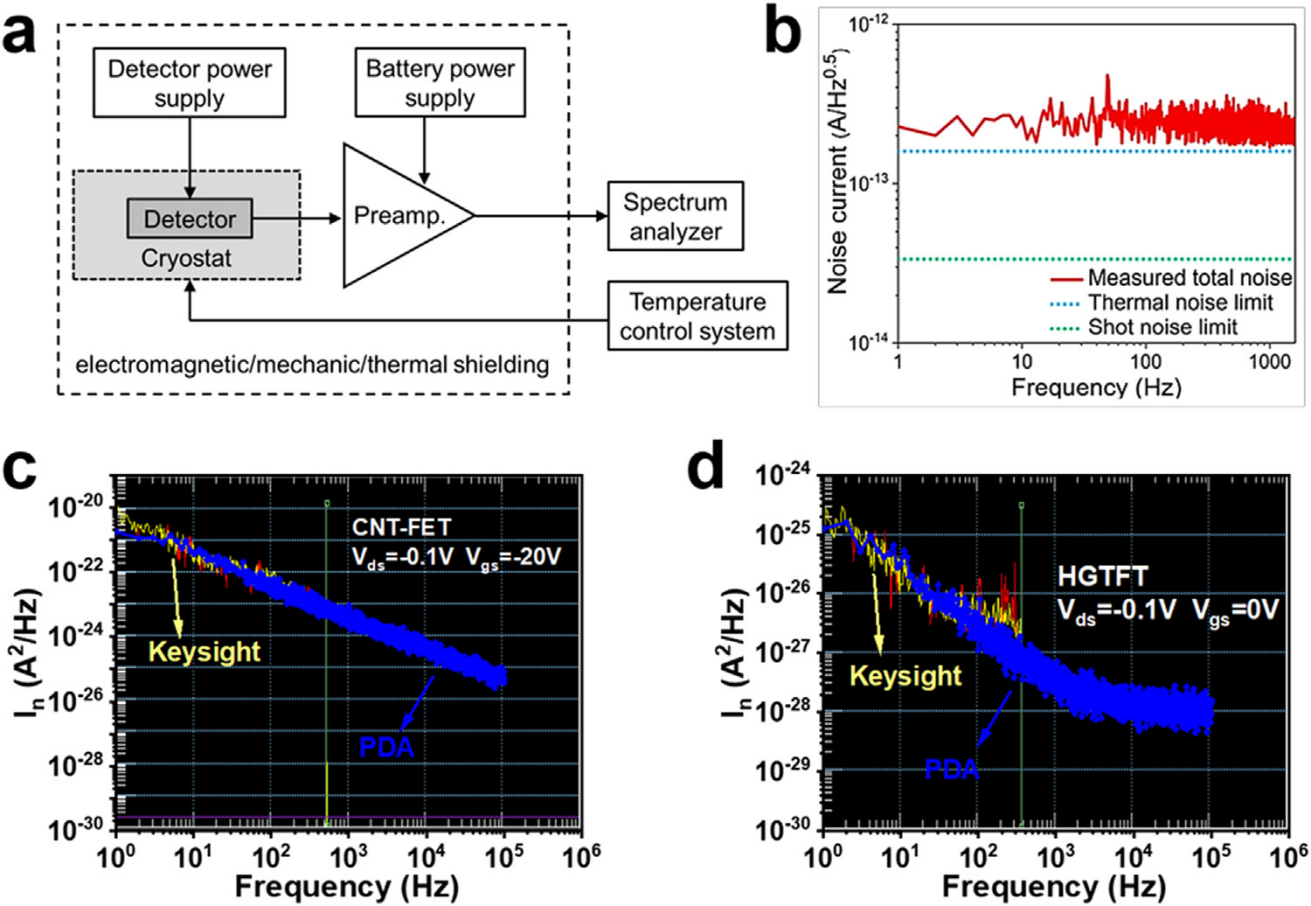
**The characterization of noise spectral density.** (a) The schematic diagram of a typical test system for the noise current measurement. Figure adapted with permission from [58], © *Polish Academy of Sciences* 2022. (b) Noise spectral density of the HgTe quantum dots photodiodes at zero bias. Reproduced with permission from Ref [59], © *American Chemical Society* 2022. (c, d) The noise power spectra of CNT phototransistors at different gate voltages, which are acquired by two different measurement systems (PDA FS380 from Bodawei Electronic Technology Co., Ltd. and E4727B from Keysight Technologies Co., Ltd.) for cross checking.

Noise is related to the randomly fluctuation of output current and voltage signals with time, which can be divided into noise current (*I_n_*) measured with current-mode readout and noise voltage (*V_n_*) measured with a voltage-mode amplifier. For high impedance CNT detectors (more than kΩ), current noise measurements are preferred. It shows the scheme of a typical noise measurement system in Fig. 5a [58]. All system elements have a significant impact on the capabilities and measurement results, including power supply, preamplifier, broadband spectrum analyzer, cable, connector, shielded housing and temperature controller. Total noise current in a detector is the sum of all the noise sources, such as thermal noise, shot noise and 1/f noise. The reliable noise characteristics effectively determine specific detectivity, which is the most important figure of merit for photodetectors.

Thermal noise (or Johnson noise) is associated with photodetector resistance. This is due to the random thermal motions of charge carriers. The magnitude of this noise current is: $\;I_{jn}={\left(\frac{4k_{B}T\Delta f}{R}\right)}^{1/2}$, where *R* is the differential resistance, and *Δf* is the electronic bandwidth of noise measurement. Shot noise comes from the statistical fluctuation of current signal caused by the flow of discrete electrons or holes. The shot noise increases with the current and obeys Poisson distribution: $I_{sn}={\left[2q(I_{d}+I_{ph})\Delta f\right]}^{1/2}$, where *I_d_* is the dark current. The 1/f noise (or flicker noise) is a type of low-frequency noise, possibly originating from the fluctuation of electron concentration. The intrinsic mechanism of 1/f noise is not completely clear, but was reported as the surface or interface states-related noise, such as generation-recombination, tunneling and diffusion [60], [61], [62]. The empirical formula of 1/f noise is: $I_{1/f}=k\frac{I^{b}}{f^{a}}\Delta f$, where *k* is a proportional constant, and *a* and *b* are related parameters. The noise sources above are independent. Thus, the total noise can be described as: $I_{n}=\sqrt{I_{jn}^{2}+I_{sn}^{2}+I_{1/f}^{2}}$.

Low-frequency noise measurements are complex and elaborate. The detector noise should be measured at its working temperature and applied bias without the influence of external disturbances. Before 2015, plenty of publications calculated the noise using the formula: $I_{n}={\left(2qI_{d}\Delta f\right)}^{1/2}$, which cannot precisely reflect the total noise of a photodetector [36,41,63]. Recently, noise current density spectra are measured by spectrum analyzers (like SR770) or integrated semiconductor parameter measurement systems (like PDA FS380 from Bodawei Electronic Technology Co., Ltd., E4727B from Keysight Technologies Co., Ltd.). It is highlighted that the dominated noise sources depend on the materials, device structure and the bias voltage. It shows the measured noise of an HgTe quantum dots-based photovoltaic device at *V*_bias_ = 0 V, indicating that the total noise comes from the shot noise and thermal noise (Fig. 5b) [59]. Whereas, the measured total noise of a CNT FET in Fig. 5c and d demonstrate the low frequency dependent 1/f noise at different gate voltages. The noise data acquired by two different measurement systems is recommended for cross verification. In addition, some publications measured the noise current by monitoring the detector current *I*(*t_j_*) values until they reached their steady-state value [64,65]. After acquiring sufficiently large number of points, the noise current *I_rms_* was calculated by the expression: $I_{rms}=\sqrt{N^{-1}\times \sum_{j=1}^{j=N}{\left[I\left(t_{j}\right)-\bar{I(t_{j})}\right]}^{2}}$, where $\bar{I(t_{j})}$ represents the steady-state average current value. This method directly shows the standard deviation of the fluctuations around *I*, but it is usually time-consuming and cannot provide the modulation frequency-dependent noise current density spectra. Currently, the detection limit of noise current by sophisticated measurement systems is ∼10 fA.

### 2.4 Noise equivalent power

The noise equivalent power (NEP) is defined as the incident power on the detector that produces a signal-to-noise ratio (SNR) of unity in a 1 Hz bandwidth. In terms of responsivity it can be written as: $\text{NEP}=\frac{I_{n}}{R_{i}}=\frac{V_{n}}{R_{v}}$ and is expressed in the spectral density unit W/Hz^1/2^. NEP can be used to determine the sensitivity of a photodetector at a given wavelength, because it is a measure of the weakest optical signal that can be detected. Using advanced methods such as lock-in detection, it is possible to detect much weaker signals and directly determine the NEP, provided that these have sufficiently low noise floor and appropriate bandwidth. Another methods to accurately determine the NEP is to measure the steady-state average photocurrent at various light intensities or the current spectral density under modulated light [66]. The intensity at which the photocurrent is equivalent to the noise floor gives a measure of the NEP. Although this is a direct methodology to obtain the accurate and effective NEP, it is challenging to accurately calibrate the incident light power at ultra-low intensities.

### 2.5 Specific detectivity

The specific detectivity (D*) is the overall figure of merit for photodetection, which is expressed in units of cm Hz^−1/2^ W^−1^, also known as Jones. The D* of a photodetector can be obtained using the following equation:

(4) $$D^{*}\left(\lambda \right)=\frac{\sqrt{A\Delta f}}{NEP\left(\lambda \right)}$$

where *A* is the effective detection area of the detector, and *Δf* is the electrical bandwidth of the test circuit. It is a comprehensive index to compare the sensitivity of two detectors.

## 3 Device architecture and detection mechanism

According to the basic light response mechanism, photodetectors based on carbon nanotubes can be categorized into thermal detectors and photon detectors. Thermal detectors involve the energy conversion from light to heat and then to electricity, while photon detectors directly convert light into electricity. Typically, thermal detectors are susceptible to slow response speeds (∼ms) and low detectivity (<10^9^ Jones), which makes it challenging for them to meet the requirements in high-end applications. In this section, we briefly review the representative works on CNTs-based thermal detectors in the past five years, and then we discuss the main device architectures and detection mechanisms of CNTs-based photon detectors.

Carbon nanotube-based thermal detectors, such as bolometers and photothermopiles, have undergone extensive study due to their unique absorption and conductivity properties. A bolometer detects changes in the temperature-dependent resistance of the suspended active materials it employs, typically requiring low thermal capacity and a high temperature resistance coefficient (TCR). CNTs can act as the nanostructured blackbody absorber with absorption coefficient higher than 95% for broadband irradiation [67], [68], [69], [70]. In 2018, Liu et al. reported an ultra-broadband bolometric photodetector based on a suspended CNT film, which showed a responsivity of 0.58 A/W and a response time of approximately 150 µs in vacuum at a 0.2 V bias [71]. Recently, Chen et al. showed the CNTs-based bolometric photodetectors with ultrahigh detectivity up to 1.35 × 10^8^ Jones and a short response time of 70 ms in air [72]. They also observed an interesting negative photocurrent owing to dominant phonon scattering transfer. Jiang et al. proposed the concept of a CNTs-based electron blackbody, which was also a good emitter with standard energy spectra at different temperatures [73]. This high-emissivity CNTs blackbody is promising in bolometric photodetection and radiometric calibration at low temperature. Photothermal devices are based on the thermoelectric effect, also known as the Seebeck effect, and can operate in the UV, visible, infrared, and THz bands. Early in 2011, St-Antoine et al. developed a CNTs-based p-n junction, which showed a thermovoltage of 16 V/K in vacuum, a response time of 36 ms, and calculated detectivity of 2 × 10^6^ Jones [74]. In 2016, He et al. reported the aligned CNTs-based photothermopiles with a responsivity of 0.1 V/W and polarization-dependent response [75]. Recently, Liu et al. reported a high-performance and broadband CNTs photothermoelectric detector with a high responsivity of 157.9 V/W, a response time of 7 ms, and a specific detectivity of 5 × 10^8^ Jones in air, demonstrating the highest overall performance among state-of-the-art ultrabroadband PDs [76]. In 2019, large-area and broadband thermoelectric infrared detection was achieved using a CNTs blackbody absorber [16]. This self-powered infrared photodetector, based on the photothermoelectric effect of a CNT forest, exhibited a 99.4% reduction in reflection, a broadband detectivity of 1.9 × 10^7^ Jones in the 2.5–25 µm spectral range, and a peak detectivity of 2.3 × 10^9^ Jones at 4.3 THz.

**Fig. 6:**
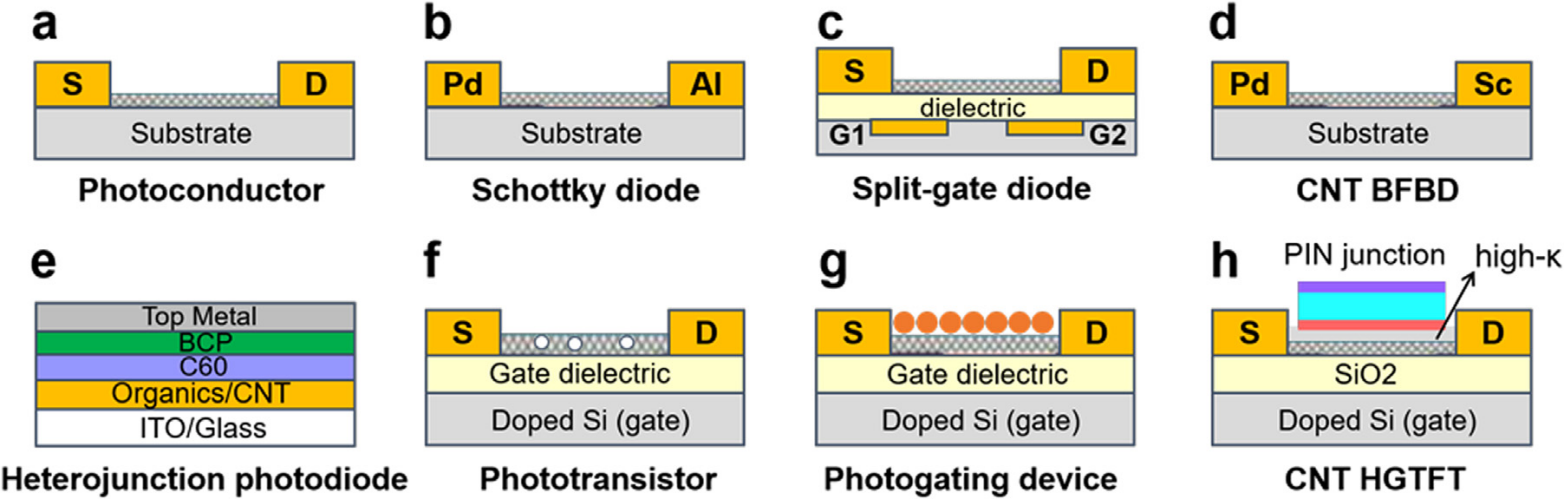
**Device architecture and configuration of CNT-based photodetectors.** (a) Schematic image depicting a simple photoconductor device architecture. (b) A Schottky-type photodiode configuration. (c) A split-gate photodiode device. (d) A bulk heterojunction blended film photodiode. (e) A barrier-free bipolar diode. (f) A bottom gate phototransistor device. (g) A hybrid-induced photogating device. (h) A PIN heterojunction-gated thin film transistor (HGTFT).

In the following section, we will discuss various types of photon detectors based on s-SWCNTs. We classify different photodetectors into three types based on the fundamental device geometry-photoconductor, photodiode and phototransistor. The device architecture and configuration of a typical device based on each class is shown schematically in Fig. 6.

### 3.1 Photoconductor

Photoconductor devices work on the principle of measuring a temporary change in conductivity of the semiconductor. The underlying mechanism is associated with the exciton creation on irradiation with light and further dissociation under an applied electric field, altering the conductivity of the semiconductor. For CNT photoconductors, a major problem is that the lack of external field manipulation limits the dark current and the efficiently separation of electron-hole (e-h) pairs.

The first experimental observation of CNT photoconductive response was reported by Fujuwara et al. at 2001 [7]. They observed two peaks in the excitation spectra around 0.7 and 1.2 eV, corresponding to the first and second gaps of semiconducting SWCNTs. In 2003, Freitag et al. demonstrated infrared laser excited photoconductivity from an individual SWCNT. Without a back-gate voltage, the observed photocurrent was around 40 pA with a wavelength range of 780–980 nm and a power of 5.6 kW cm^−2^ [77]. Later, numerous studies focus on the time-resolved photocurrent of individual CNT devices to investigate the photophysics of nanomaterial-based device, but unfortunately they all showed weak photocurrent signal and did not estimate the responsivity or detectivity [78,79]. Some reports tried to improve the light absorption by using aligned or random-oriented CNT thin films. Wang et al. synthesized horizontally aligned SWCNTs suspended across silicon trenches (40 µm) and fabricated photoconductors with Al electrodes [80]. The resistance dropped by 22.86% under infrared illumination with power less than 4 mW, and the response time was about 500 µs. Whereas, the low density of CNTs (8–20 CNTs/device) still limit the absorption cross section. Additionally, many reports of photodetectors based on CNT films exhibited mixed mechanisms of the photoconductive effect and the thermal effect, especially when metallic CNTs existed. In 2012, Wu et al. achieved the photoconductivity enhancement by incorporating CNTs with polymers [81]. The near-infrared detectivity of the device was 2.3 × 10^8^ cm Hz^1/2^ W^−1^, and the rise and fall times were 0.6 and 1.4 ms, respectively. Compared with thermal detectors, the enhanced D* and speed were ascribed to the enhanced local electric field around the CNTs, facilitating the exciton dissociation. Later studies found that graphene and other types of polymers can also form bulk heterojunctions with CNTs, with which an infrared detectivity lower than 10^9^ cm Hz^1/2^ W^−1^ was obtained [41,82,83].

### 3.2 Photodiode

The key core of a photodiode is the built-in potential, which is critical to separate photo-induced excitons or e-h pairs for converting the light signal to photocurrent. The typical CNT-based photodiodes include Schottky diode, split-gate diode (PIN type), barrier-free bipolar diode (BFBD) and vertical heterojunction diode. Unlike traditional Si or InGaAs photodiodes used under reverse bias, most reported CNT diodes operated at zero bias for low dark current, also referred to as the self-powered photodetectors.

**Fig. 7:**
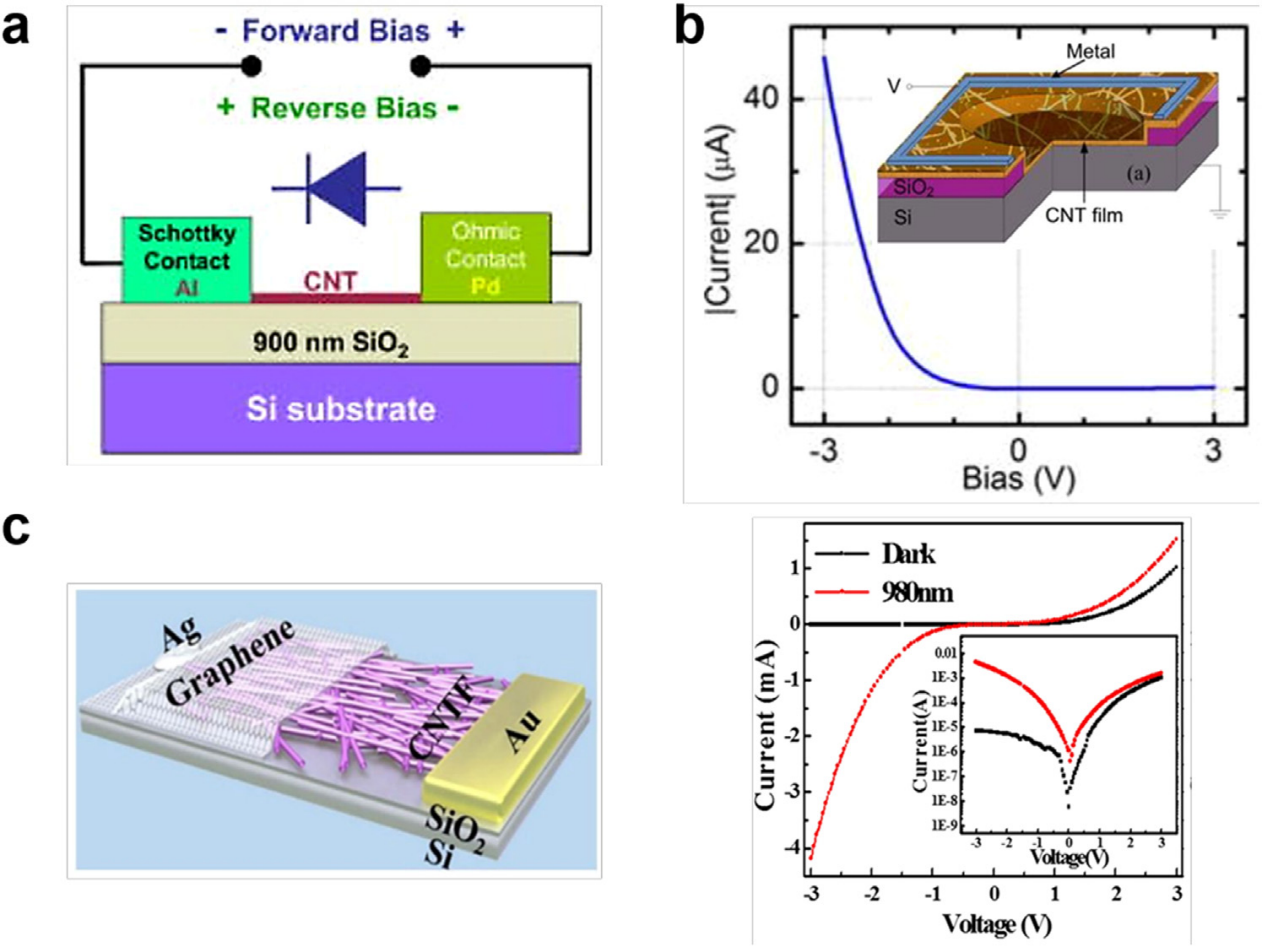
**Structures and electronic characteristics of distinct CNT-based Schottky diodes.** (a) Pd-Al contacted CNT diode. Reproduced with permission from Ref [84], © *American Institute of Physics* 2005. (b) s-SWCNT film/p-type silicon Schottky diode with vertical structure. Reproduced with permission from Ref [86], © *AVS AMER INST PHYSICS* 2012. (c) Graphene/s-SWCNTs film Schottky diode (left) and corresponding output characteristics (right). Reproduced with permission from Ref [87], © *Springer Nature* 2016.

Schottky-type photodiode. By changing the work function of the metal, CNT-based Schottky diodes can be constructed. The metal/semiconductor interface provides a potential barrier that separates the optically generated e-h pairs. In 2005, Hasko et al. demonstrated a CNT based Schottky diode by using a Pd contact (high-work-function metal) and an Al contact (low-work-function metal) at the two ends of a single-wall CNT, as shown in Fig. 7a [84]. The device showed a rectification ratio of 10^3^ at a high back gate voltage V_g_ = 10 V. In 2010, Yang et al. reported the symmetric Au-CNT-Au, Ag-CNT-Ag and asymmetric Ag-CNT-Au Schottky diodes [85]. The two Schottky barriers in asymmetric devices can efficiently suppress dark current and increase photocurrent, giving rise to a high open-circuit voltage of 0.45 V. As shown in Fig. 7b, Ant et al. fabricated the vertical geometry SWCNT film/*p*-type silicon Schottky diode, where the CNT film acts as the transparent metal [86]. The CNT film-Si Schottky barrier photodetectors exhibited a high responsivity of 0.10 A/W, NEP of 1.4  ×  10^−10^ W and D* of 1.1 × 10^9^ cm Hz^−1/2^ W^−1^ at 1 V bias under 840 nm illumination. In Fig. 7c, Luo et al. presented a broadband photodetector based on CNT film/graphene Schottky junction [87]. The as-fabricated device exhibited peak sensitivity at 600 nm and 920 nm with a fast response speed (τ_r_ = 68 µs, τ_f_ = 78 µs), high on-off ratio of 10^2^, responsivity of 209 mAW^−1^ and detectivity of 4.87  ×  10^10^ cmHz^1/2^W^−1^, due to the high quality of the CNT/graphene interface. In 2018, An et al. also reported the SWCNT/graphene Schottky diode based on a double-layer stacked heterostructure [37]. The photoconductive gain *G* was speculated on the order of ∼10^4^, and the peak responsivity at 532 nm was 78 A/W. In the Schottky junction photodetector, the intrinsic photoelectric properties of SWCNTs can be maintained, but the large dark current at a certain bias and low EQE limit the detectivity.

**Fig. 8:**
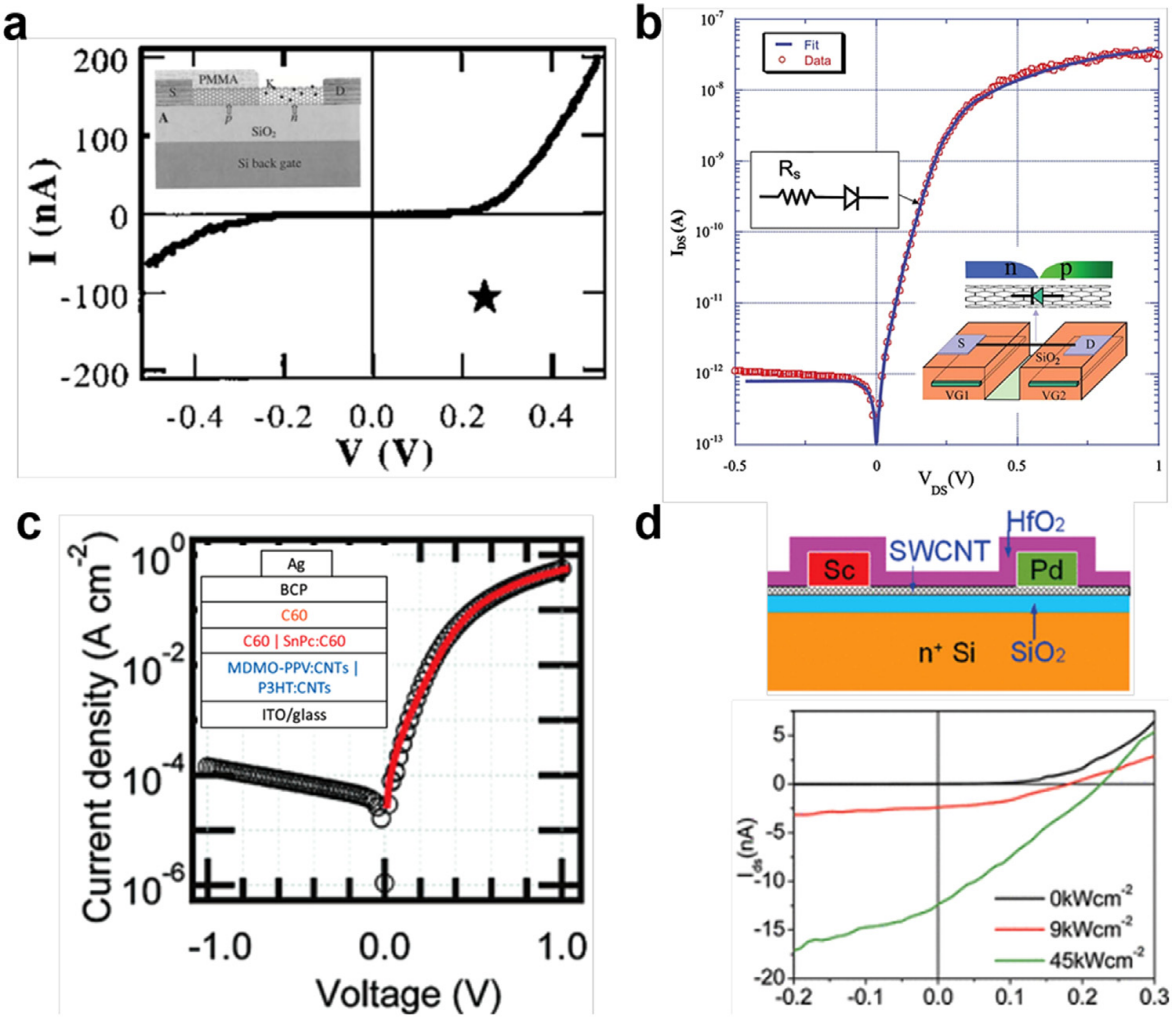
**Structures of distinct CNT PN junction diodes.** (a) Chemical doping CNT diode and corresponding output characteristics. Reproduced with permission from Ref [91], © *American Association for the Advancement of Science* 2000. (b) Split-gate CNT diode. Reproduced with permission from Ref [92], © *American Institute of Physics* 2005. (c) Vertical heterojunction CNT diode. Reproduced with permission from Ref [36], © *American Chemical Society* 2009. (d) Asymmetrically contacted or barrier-free-bipolar-diode based on CNT (top) and corresponding output characteristics (bottom). Reproduced with permission from Ref [94], © *American Chemical Society* 2009.

Split-gate photodiode. CNT p-n diode was first made by using chemical doping method [88], [89], [90], such as K atom doping and polymer doping shown in Fig. 8a [91]. However, the inserted dopants into the sp^2^ lattice of CNTs will break the perfect intrinsic structure, lead to larger scattering and thus lower the device performance. Based on the principle of electrostatic doping, SWCNT split-gate photodiode can be constructed with ideal behavior. Different bias polarities on the split gate electrostatically couple to form separate regions of electron and hole doping along the SWCNT, enabling to study the intrinsic properties of CNT without introducing band gap changes in the doped state. In 2005, Lee fabricated a split-gate SWCNT diode with a good rectification of 10^4^–10^5^ and an ideal factor of 1.2–1.3, as shown in Fig. 8b [92]. Under illumination, SWNT diodes showed significant power conversion efficiencies with extrapolated V_oc_ > 0.8 V and FF > 0.8, owing to enhanced properties of an ideal diode. In 2013, Mizutani et al. reported an ultra-low leakage current CNT diode with split-gate and asymmetric contact geometry [93]. The device displayed strong rectification, with a leakage current of 2 × 10^−14^ A and an ideality factor of 2.01.

Heterojunction diode. As the detectors often suffer from the low densities of SWCNTs in the device channel and low EQE, a more efficient device architecture was developed by designing planar SWCNT/polymer heterojunctions. The energy band alignment at the SWCNT/polymer interface facilitate the electron-hole separation, leading to enhanced responsivity. In 2009, Forrest et al. first reported a CNT/C_60_ heterojunction diode, as shown in Fig. 8c [36]. Polymer-wrapped HiPco s-SWCNTs and C60 formed the type II heterojunction, showing a broad spectral response (400–1450 nm), a response time of ∼7.2 ns, and a specific detectivity of > 10^10^ cm Hz^1/2^ W^−1^. Later, Arnold et al. studied the exciton dissociation and interfacial charge transfer from SWCNT films to a variety of polymeric photovoltaic materials [17]. The results showed that sufficient band offsets for dissociation occurred when SWCNTs were paired with C_60_, [C_61_]-PCBM, P3OT and P3HT. Typically, the separated photoelectrons were transferred from the SWCNT layer into the C_60_ layer, giving an open-circuit photovoltage and short-circuit photocurrent. Later works showed the performance of CNT heterojunction diodes can be further improved by proper choice of CNT chirality and removing the wrapped molecules around CNTs [35,95,96].

Barrier-free bipolar diodes (BFBD). With perfect n-type (Sc or Y) and p-type (Pd) Ohmic contacts, SWCNT BFBD can be constructed. In this device geometry, both electrons and holes can be efficiently injected from the asymmetrically contacted electrodes into the conduction and valance bands of SWCNTs, due to the absence of dopants-induced scattering and barrier-blocked charge transport. Thus, BFBD devices can guarantee the perfect structure of intrinsic CNTs and provide a possibly maximum photovoltage. In 2009, Peng et al. reported the first BFBD detector with Pd/Sc asymmetric contacts, as shown in Fig. 8d [94]. The device with the channel length of 800 nm showed a good ideal factor of about 1.1 to 1.3, large open-circuit voltage of 0.23 V and photocurrent of more than 15 nA. Later work with SWCNT arrays showed improved responsivity of 9.87 × 10^−5^ A/W and infrared spectral detectivity of 1.09 × 10^7^ cmHz^1/2^/W [97].

In 2011, Peng et al. demonstrated that the photovoltage of CNT BFBD can be readily multiplied by using virtual contacts [98]. The virtual contacts can be fabricated simply by depositing two different metals (Pd and Sc) together. In this type of cascade detectors, the virtual contacts create separated electron and hole regions and offer internal parallel paths for the current, leading to the photovoltage multiplication and significant improvement on the signal-to-noise ratio. They observed photovoltage of more than 1.0 V from a 10-µm-long CNT with the single-cell photovoltage of ∼0.2 V. In 2016, they achieved a broadband infrared CNT BFBD photodetector by using high purity (> 99.9%) SWCNT networks. The devices showed a peak voltage responsivity of 1.5 × 10^8^ V/W and a high D* up to 1.25 × 10^11^ cm Hz^−1/2^ W^−1^.

**Fig. 9:**
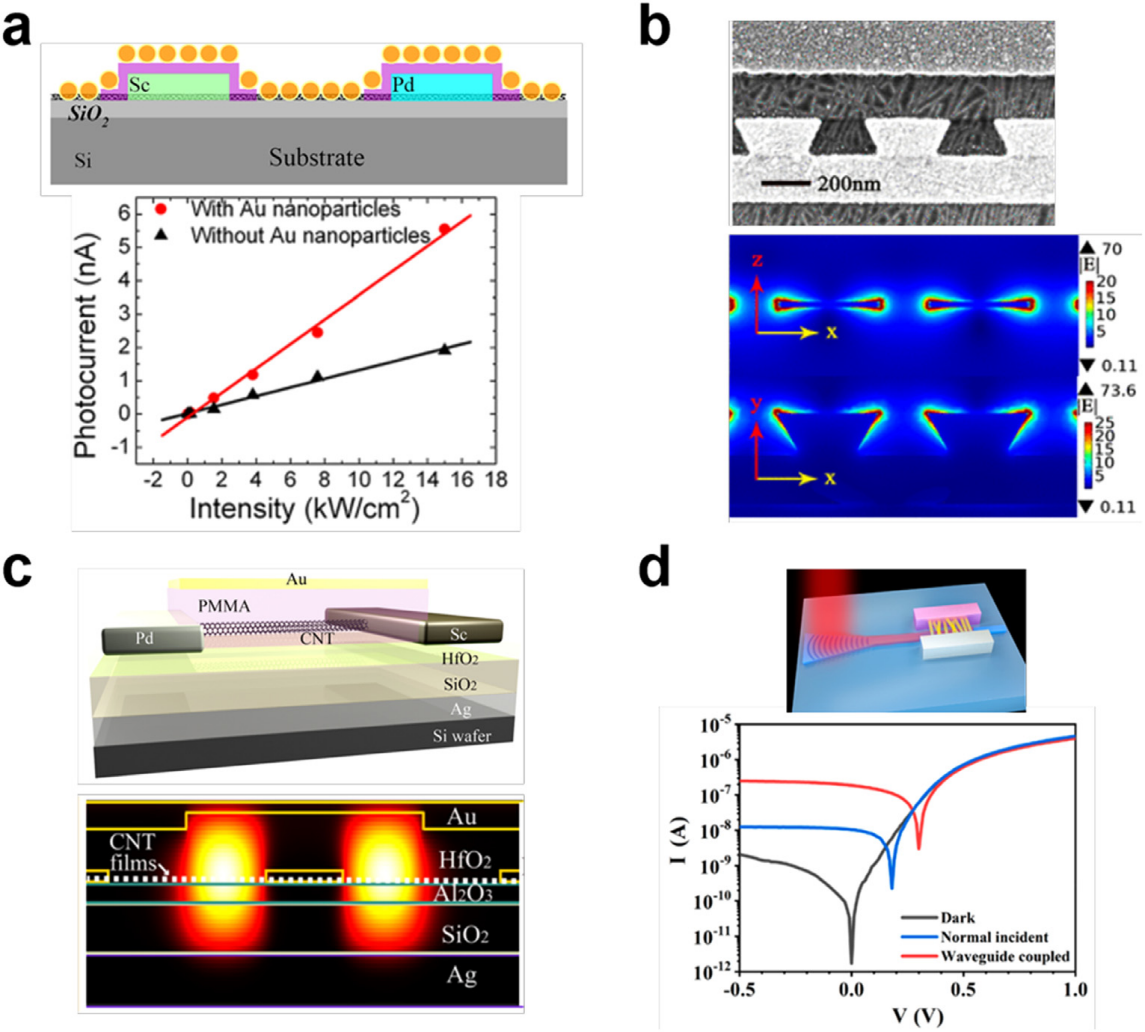
**Different optical manipulations for CNT BFBD devices.** (a) Plasmonic near-field coupling between the CNT and Au nanoparticles. Reproduced with permission from Ref [99], © *American Institute of Physics* 2013. (b) Optical manipulation structure of axe-like plasmonic contact electrode. Reproduced with permission from Ref [101], © *American Chemical Society* 2017. (c) Optical manipulation structure of microcavity. Reproduced with permission from Ref [102], © *American Chemical Society* 2016. (d) Waveguide-integrated BFBD photodetector. Reproduced with permission from Ref [103], © *American Chemical Society* 2020.

In spite of these advances, the small absorption cross-section of individual CNTs or low-density CNT monolayers (absorption < 2%) always limit the detector performance. Optical manipulations for CNT diodes were thus investigated, which included localized surface plasmons (LSPs), surface plasmon polaritons (SPPs), resonant cavities, and waveguides. In 2013, a plasmonic nanostructure of self-assembled Au nanoparticles array was coupled with CNT BFBD, as shown in Fig. 9a [99]. More than 3 times photocurrent enhancement was observed, attributed to the strong plasmonic near-field coupling with CNTs. An axe-like plasmonic contact electrode also facilitated considerably stronger field for efficient light absorption and collection of carriers in CNT BFBD (Fig. 9b) [100,101]. The largest enhancement was 200 times at the gate voltage of 0 V for a wavelength of 2000 nm. In 2016, the cavity-integrated chirality-sorted CNT BFBD was achieved, showing a higher suppression ratio, photocurrent of 5 pA, and the spectral full width at half-maximum of 33 nm at a signal wavelength of 1200 nm, as shown in Fig. 9c [102]. Peng et al. also reported the silicon-waveguide-integrated CNT BFBD infrared detector and monolithic optoelectronic system [103]. The waveguide-integrated photodetectors exhibited 12.5 mA/W photoresponsivity at 1530 nm, identifying an enhanced absorption efficiency by 97.6 times compared to that without the waveguide (Fig. 9d) [103]. The absorptivity can be significantly increased by these optical manipulation strategies, but the conversion efficiency (from excitons to photocurrent) is still low because of the weak build-in electric-field in CNT BFBD. In 2023, Wang et al. used stacked CNT films and contact electrode design to solve this problem [104]. When the thickness of network CNT film reached 5 nm, the Pd/Hf contacted CNT BFBD device showed a maximum responsivity of 120 mA/W at 1800 nm wavelength, a linear dynamic range over 118 dB, a peak detectivity of 3.94 × 10^9^ cm Hz^−1/2^ W^−1^ at 300 K and over 2.27 × 10^11^ cm Hz^−1/2^ W^−1^ at 180 K. This work demonstrates the potential of the CNT film for future infrared imaging, whereas the fabrication process is extremely complex.

### 3.3 Phototransistor

**Fig. 10:**
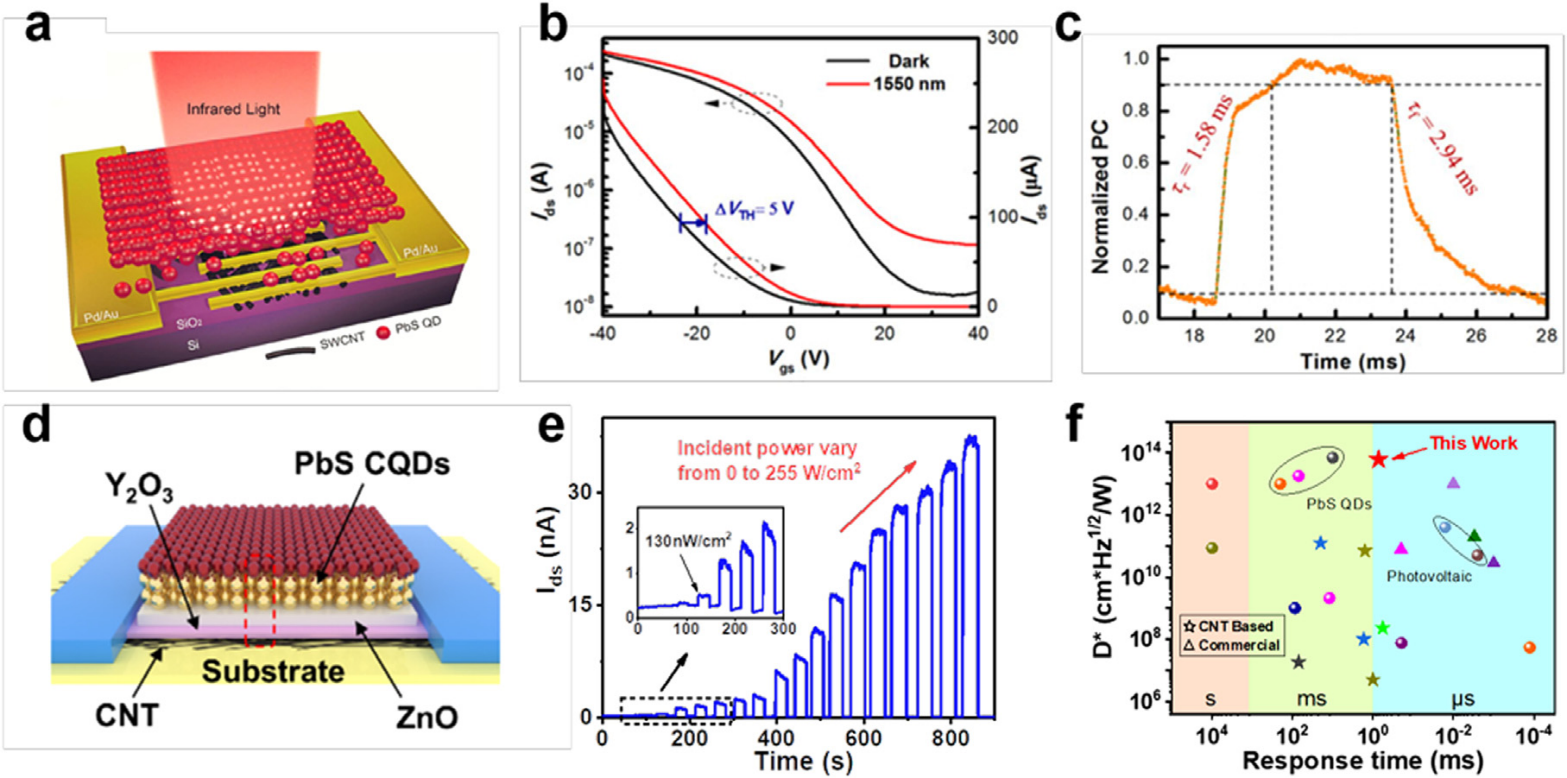
**Typical CNT-based phototransistors.** (a) Schematic diagram of the photogating transistor structure. (b) Transfer characteristics of the hybrid CNT/PbS QDs photogating transistor. (c) Response time of the photogating transistor. Reproduced with permission from Ref [105], © *Institute of Electrical and Electronics Engineers Inc.* 2018. (d) Schematic diagram of the CNT HGTFT structure. (e) Time-resolved photoresponse of the CNT HGTFT. (f) Performance benchmark. Reproduced with permission from Ref [106], © *American Institute of Physics* 2022.

Phototransistor is a three-terminal device, in which the gate voltage provides a greater degree of control over the conductivity of the semiconductor. The application of a gate bias can be used to control the position of the Fermi level and change the carrier density in the active materials. Besides, phototransistor can be operated as a switch or an amplifier, holding great potential in optoelectronic computing and high-performance photodetection. Typical CNT-based phototransistors include hybrid photogating transistor and heterojunction gated thin-film transistors (HGTFT), as shown in Fig. 10.

Hybrid photogating transistor. Photogating is considered as a way of conductance modulation through trap- or hybrid-induced gate voltage, which is usually observed in low dimensional photodetectors [107,108]. In a photogating transistor, one type of the photogenerated carriers is trapped and they have a certain spatial distribution, producing an additional electric field like gate voltage to modulate the channel conductance. Thus, most photogating-dominated photodetectors show high gain (*τ*/*τ*_T_) and limited response speed because of the prolonged releasing process of trapped carriers for recombination. For phototransistor based on single semiconductor channels such as CNT films, a trade-off between the absorption and the amplification process exists within the same channel. The atomically thick CNT monolayers allow efficient field-effect modulation and low EQE, while the thicker CNT films behave in the opposite manner. To overcome this trade-off, an encouraging method is to sensitize the thin channel materials with strongly absorbing semiconductors (sensitizers) of opposite doping polarity to create the hybrid photogating transistor.

In 2012, Koppens et al. reported a hybrid graphene/PbS nanocrystals phototransistor, fabricated using mechanically exfoliated single/bilayer graphene flakes and layer-by-layer assembled PbS QDs [64]. In this device, PbS QDs act as the strongly light-absorbing and spectrally-tunable layer to achieve a high EQE. Photoexcited holes in the PbS QDs are transferred to the graphene layer and drift at a certain voltage bias V_ds_, while photoexcited electrons remain trapped in the PbS QDs leading to the photogating effect. They obtained ultrahigh gain of 2 × 10^8^, a responsivity of ∼10^7^ A W^–1^ and a detectivity > 10^13^ cm Hz^−1/2^ W^−1^. However, the hybrid device shows a slow temporal response with a rise time of ∼10 ms and a bi-exponential decay of ∼100 ms and ∼2 s. Later, numerous works focused on this type of photodetector, but few had surpassed this detectivity or overcome the bottleneck of gain-bandwidth trade-off [31,33,42,43,109,110]. In 2012, CNT/TiO_2_ core-shell nanowire-based phototransistor was reported with high photogain of 1.4 × 10^4^ and response/recovery time of 4.3/10.2 ms in the UV–visible detection range [111]. Bao et al. fabricated a SWCNT/C60 hybrid photogating transistor with improved responsivity of ∼80 A/W and detectivity of 1.17 × 10^9^ cm Hz^−1/2^ W^−1^ (obtained at 1000 Hz) at 1200 nm wavelength [40]. In Fig. 10a-c, Hu et al. demonstrated the hybrid SWCNTs/PbS QDs phototransistor, in which the photogenerated holes inject into SWCNTs and electrons trapped in PbS QDs film. The device achieved a high response rate of 353.4 A/W, specific detectivity of 7.1 × 10^10^ cm Hz^−1/2^ W^−1^, and response time of 1.58 ms [105]. In 2020, giga-gain exceeding 10^9^ at room temperature in CNT/P3HT hybrid phototransistor was achieved under 530 nm illumination, due to the trapping of negative charges near the nanotubes [30]. This mechanism led to an integrating detector that was shown to detect as little as 490 aW and to resolve as few as 8 − 13 photons/nanotube at room temperature. Sun et al. then reported a hybrid phototransistor based on CNT network and all-inorganic perovskite CsPbBr_3_-QDs, showing a high responsivity of 5.1 × 10^7^ A/W and a specific detectivity of 2 × 10^16^ cm Hz^−1/2^ W^−1^ at 516 nm wavelength [26]. They also presented a flexible optoelectronic sensor array of 32 × 32 pixels for the human eye inspired neuromorphic vision systems. However, all the CNT-based photogating transistors have showed slow response speed (τ_r_ and τ_f_ > 1 ms) until now without an additional gate pulse to reset the trapped carriers. In 2017, Adinolfi and Sargent reported a silicon/PbS QDs-based photovoltage field-effect transistor with high gain (G_P_ > 10^4^), high speed (100 kHz) and thus a large G_P_ × BW product (10^4^ × 10^5^ s^−1^) at 1500 nm wavelength [112]. This device is still a photogating-dominated transistor limited by the trapping-related mechanism, in which the photoelectric transduction (from light to electrons or holes) and charge transport are mixed in the active channel. The fast response is probably attributed to the low defects density in PbS QDs and excellent Si/PbS QD interface.

CNT-based HGTFT. The schematic structure of a heterojunction-gated TFT is shown in Fig. 10d-f. In 2022, Zhang et al. designed the SWCNT HGTFT by using the PbS QD/ZnO PN junction as the photosensitizer, an insulating high-κ Y_2_O_3_ layer as the gate dielectric, and the underlying CNT network as the charge transport layer [106]. In this device, the PN junction of ∼250 nm achieved high EQE and a large light-dependent open-circuit voltage V_oc_. Meanwhile, the CNT TFT facilitated the amplification of V_oc_ signal through the electrostatic-coupling mechanism. Unlike the photogating transistor, the sandwiched Y_2_O_3_ dielectric between the PN junction and the CNT channel enables the decoupling of the photoelectric conversion (from light to V_oc_ in the photosensitizer) and charge transport (form V_oc_ to photocurrent in the CNT channel), which can avoid mutual interference and give rise to both enhanced sensitivity and fast response. The fabricated HGTFT-based infrared detector demonstrated a high responsivity of 1.65 × 10^4^ A/W, a fast response of 570 µs and excellent specific detectivity of 5.6 × 10^13^ cm Hz^−1/2^ W^−1^ at 1300 nm wavelength. In 2023, Li et al. reported a negative photoconductance (NPC) HGTFT device with a hybrid PM6/Y6 heterojunction deposited on the CNTs channel [113]. With the in-situ signal amplification, this photodetector showed a controllable NPC effect, a high responsivity of 72.6 A/W at 880 nm, and fast NPC response/recovery of 7 ms and 5 ms, respectively. They also observed the adjustable gate switching between NPC and positive photoconductance (PPC) and demonstrated the capability of HGTFT in novel in-sensor computing at the device level. Therefore, this CNT HGTFT may represent a universal architecture for highly sensitive low-dimensional semiconductor based infrared photodetectors.

## 4 Novel applications

SWCNTs have the advantages of high carrier mobility, low intrinsic capacitance, good mechanical strength, high thermal stability and excellent radiation tolerance, offering a powerful platform for electronic and optoelectronic devices [114], [115], [116], [117], [118], [119]. The bandgap *E*_g_ of SWCNTs is diameter- and chirality- tunable across a wide range of energies from the ultraviolet to the infrared. In this section, we demonstrate novel optoelectronic applications based on CNT photodetectors, including polarization-sensitive photodetection, optical synapses, image sensing, optical communications and optoelectronic integration.

**Fig. 11:**
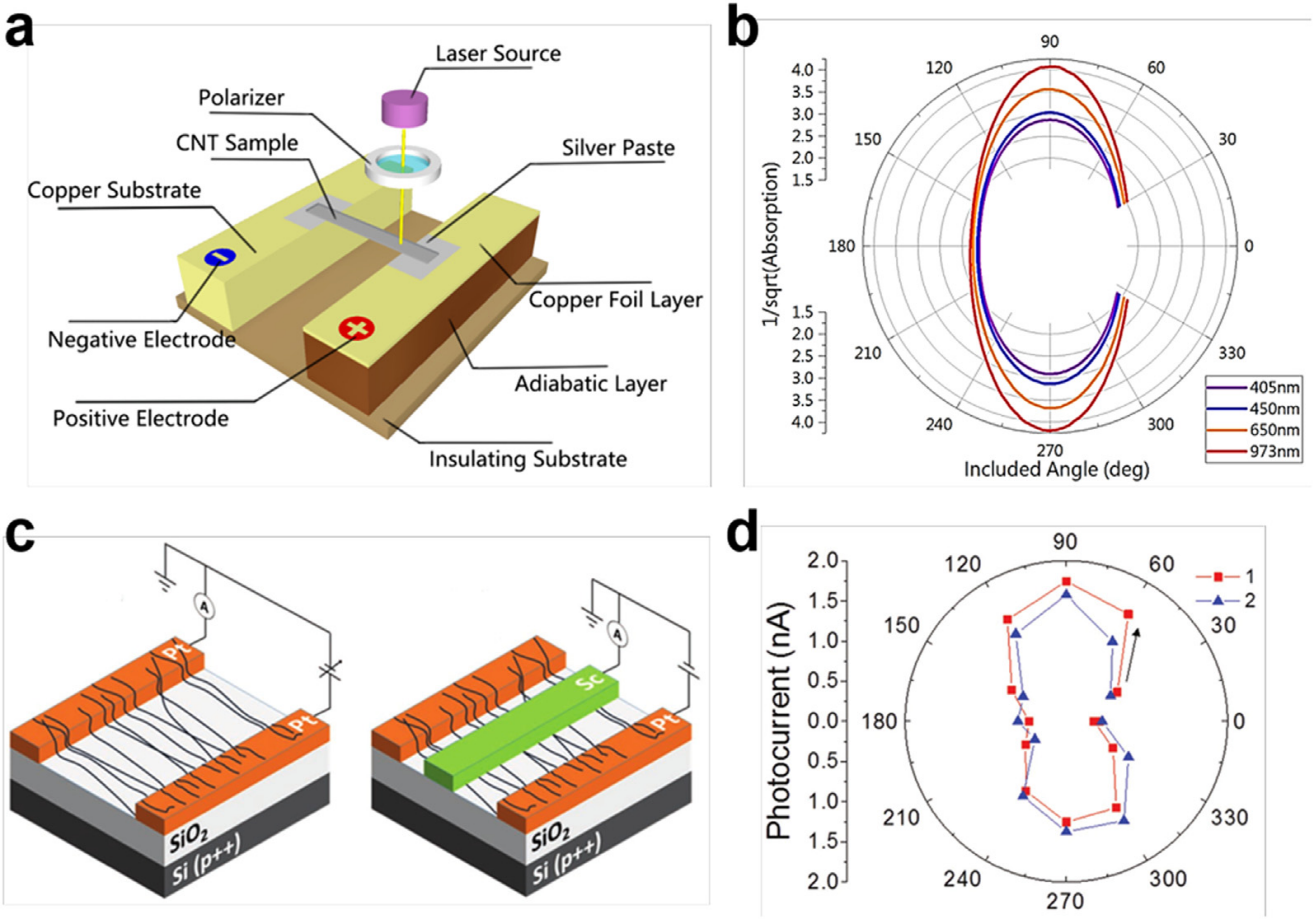
**Polarization-sensitive CNT photodetectors.** (a) Schematic illustration of a polarized photodetector based on aligned CNTs. (b) The angle-dependent absorption of the photodetector. Reproduced with permission from Ref [125], © American Chemical Society 2016. (c) Illustration of the cross-sections of aligned SWNT transistors with the method of dielectrophoresis. (d) The polarization-denpendent photocurrent at zero bias. Reproduced with permission from Ref [126], © Wiley-VCH Verlag 2017.

Polarization-sensitive photodetection. Due to the constrained electron motion and electron-phonon coupling in 1D geometry, CNT is a promising material for polarization-dependent absorption and detection [120], [121], [122]. In 2011, Jiang et al. fabricated a polarized infrared thermal detector based on aligned multiwalled CNT films [123]. This CNT sensor showed a responsivity of 30 V/W and a response time of 4.4 ms. In 2016, Lee et al. reported the enhanced anisotropic terahertz response of freestanding multiwalled CNTs, attributing to the strong nonlinear electron dynamics in CNTs [124]. Fan et al. also reported the aligned CNT-based polarized photodetector and showed the experiments and mathematical analysis of anisotropic optical response to different wavelength, as shown in Fig. 11a and b [125]. The absorption of polarized light between parallel and perpendicular configurations is related to the thermoelectric effect due to the conductive nature of CNTs. However, the chiral mixing of synthesized CNTs and the need for precise positioning of CNTs-based devices hinder the development of devices for practical applications. In 2017, Filoramo et al. achieved high-purity sorting and local deposition of aligned CNTs by combining a polymer-assisted extraction method with dielectrophoresis (DEP), as shown in Fig. 11c [126]. This method enabled the selection of SWCNTs with a diameter of 1–1.2 nm and the efficiently deposition of aligned array between prepatterned electrodes. Using Pt/Sc asymmetric contacts, each device can operate as a photoemitter and as polarization-sensitive photodetector in the telecom band 1.55 µm in air at room temperature, as shown in Fig. 11d. In 2019, Jiang et al. reported a self-assembly method to achieve highly aligned and uniform SWCNTs film in wafer scale, and they fabricated the N_2_H_4_-doped SWCNTs-Au heterojunction-based broadband photodetectors [25]. The device showed a maximum responsiveness of 292.5 mA/W and a built-in polarimeter with a linear dichroism of approximately 1.6.

Optical synapses. With increasing demands for handling “big data” nowadays, novel computing systems based on the non-von Neumann architecture have been rapidly developing, leading to the exploration of deep learning and artificial intelligence (AI) with lower power consumption. For example, autonomous vehicles require light-controlled reconfigurable optical synapse for image recognition, wherein the artificial devices are able to learn, memorize, and process visual perceptions like a human retina. Optoelectronic synapse combines a photodetector and an electronic synapse in a single device platform, which typically exhibits multiple nonvolatile conductance states when subjected to light pulses-analogous to a photodetector with memory capability. Optoelectronic synaptic devices based on low-dimensional materials can be constructed by using functional oxides, heterojunctions and ferroelectric dielectric [127]. Early in 2017, Zhang et al. reported a graphene/CNT hybrid phototransistor that can respond to optical stimuli in a highly neuron­like fashion and exhibit flexible tuning of both short­ and long­term plasticity [128]. They also showed advanced optical spike processing including NOR and AND/OR logic operations, providing an important foundation for the next generation photonics­enabled neuromorphic sensing and computing. Then, CNT photonic synaptic behaviors were studied with organic/CNT or perovskite/CNT hybrids [26,[129], [130], [131], [132]]. Typically, Huang et al. reported the high-performance infrared phototransistors with ternary semiconductors of lead-free perovskite/SWCNT in 2020 and organic/SWCNT in 2021, respectively [22,23]. Due to the internal gain, both of the hybrid devices showed high responsivity (10^4^–10^6^ A/W), a large I_photo_/I_dark_ ratio and ability to detect ultra-weak infrared light intensity of 100 nW cm^−2^. The devices exhibited good operational stability under continue cycling test and were employed as synaptic devices to simulate the processing information under visible light signal. Zhao et al. also reported an artificial synaptic device based on the honey-CNT memristor, with which synaptic plasticity of biological neurons, spatial summation and shunting inhibition were emulated [133]. In this device, the memristive behaviors were explained by the electrochemical formation and dissolution of conductive paths in the honey-CNT film. Despite the rapid progress in exploring the projected promise of CNTs for artificial synapses, challenges still remain in controlling the charge trapping/detrapping process, improving the materials uniformity, reducing the device energy efficiency and enhancing the component integration density.

**Fig. 12:**
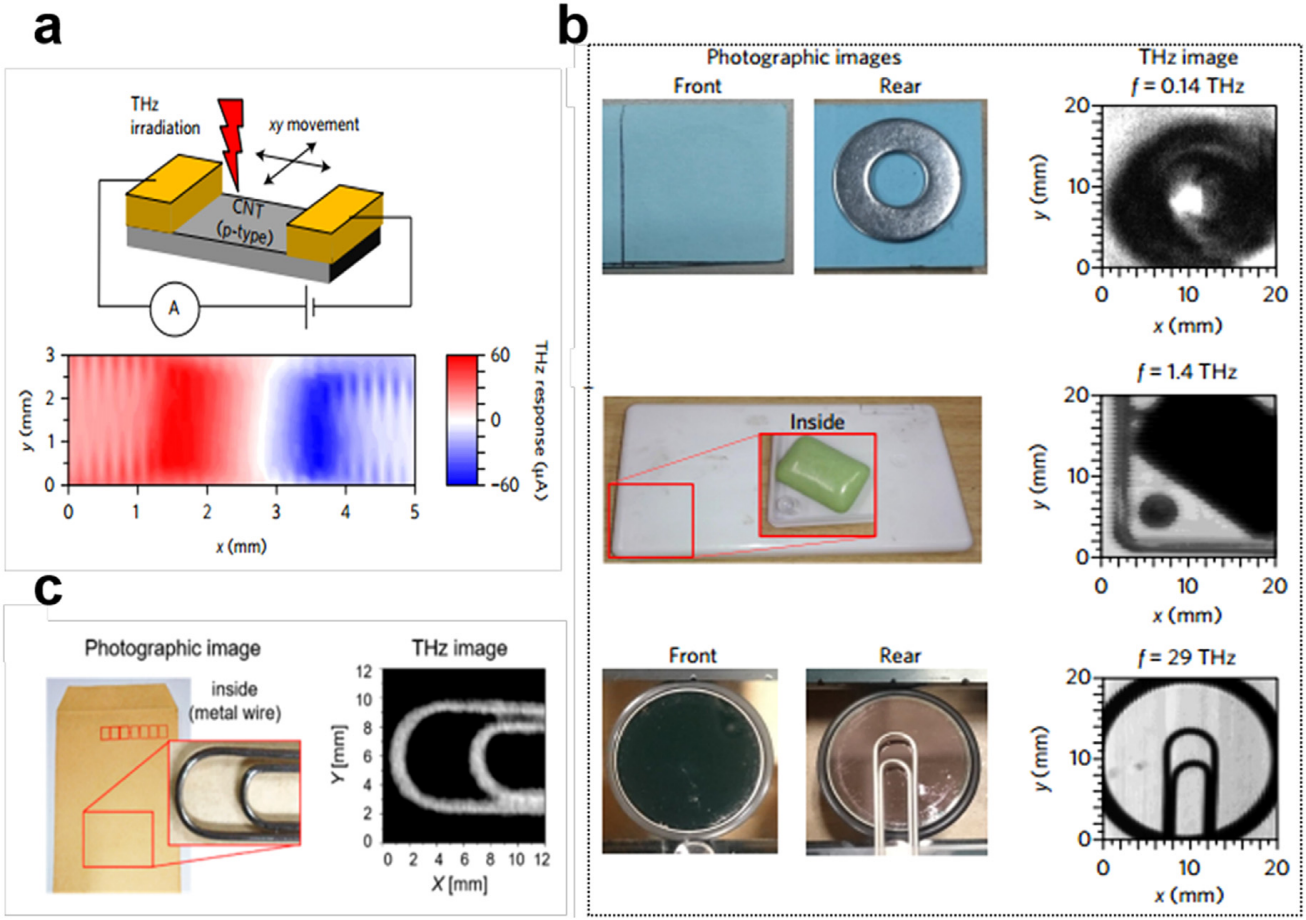
**CNTs-based THz imager.** (a) THz response map obtained by scanning the THz laser spot at 29 THz. (b) THz imaging of samples concealed behind opaque objects. Reproduced with permission from Ref [134], © *Springer Nature* 2016. (c) Photographic image of the envelope and THz image acquired by the CNT THz imager. Reproduced with permission from Ref [135], © *American Chemical Society* 2018.

Image sensing. Image sensors are of use in a wide range of fields including machine vision, security monitoring, bioimaging and military. The solution-processed CNT film enables low-temperature processing for fabrication, facilitating the direct integration of detector arrays with Si readout circuits. However, it is challenging to fabricate an image sensor, which necessitates the scalable fabrication of detectors, the addition of a readout circuit, and the compatible integration process of the two. In 2016, Suzuki et al. reported a wideband, flexible and portable terahertz (THz) scanner based on an array of CNTs devices, which realized room-temperature terahertz detection in a wide frequency band from 0.14 to 39 THz, as shown in Fig. 12a, b [134]. The device successfully conducted active imaging of flat and curved samples, multi-view scanning of cylindrical samples, and passive wearable imaging of human hands. This present technology paves the way for the development of real-time medical monitoring and non-invasive inspections without the need for complicated optical components and systems. Later in 2018, a bendable THz imager based on CNTs films was reported by the same group (Fig. 12c) [135]. Utilizing the electronic double-layer technique with ionic liquids, the performance of the THz detector was improved, resulting in an on/off resistance ratio of 2758. Additionally, gate doping was optimized to tune the Fermi level of CNTs. Using this imager, the researchers successfully visualized a metal envelope inside a standard envelope. In this field, the design and fabrication of universal readout circuits are crucial for accelerating the development of image sensors, and collaborative efforts among research groups are also required.

**Fig. 13:**
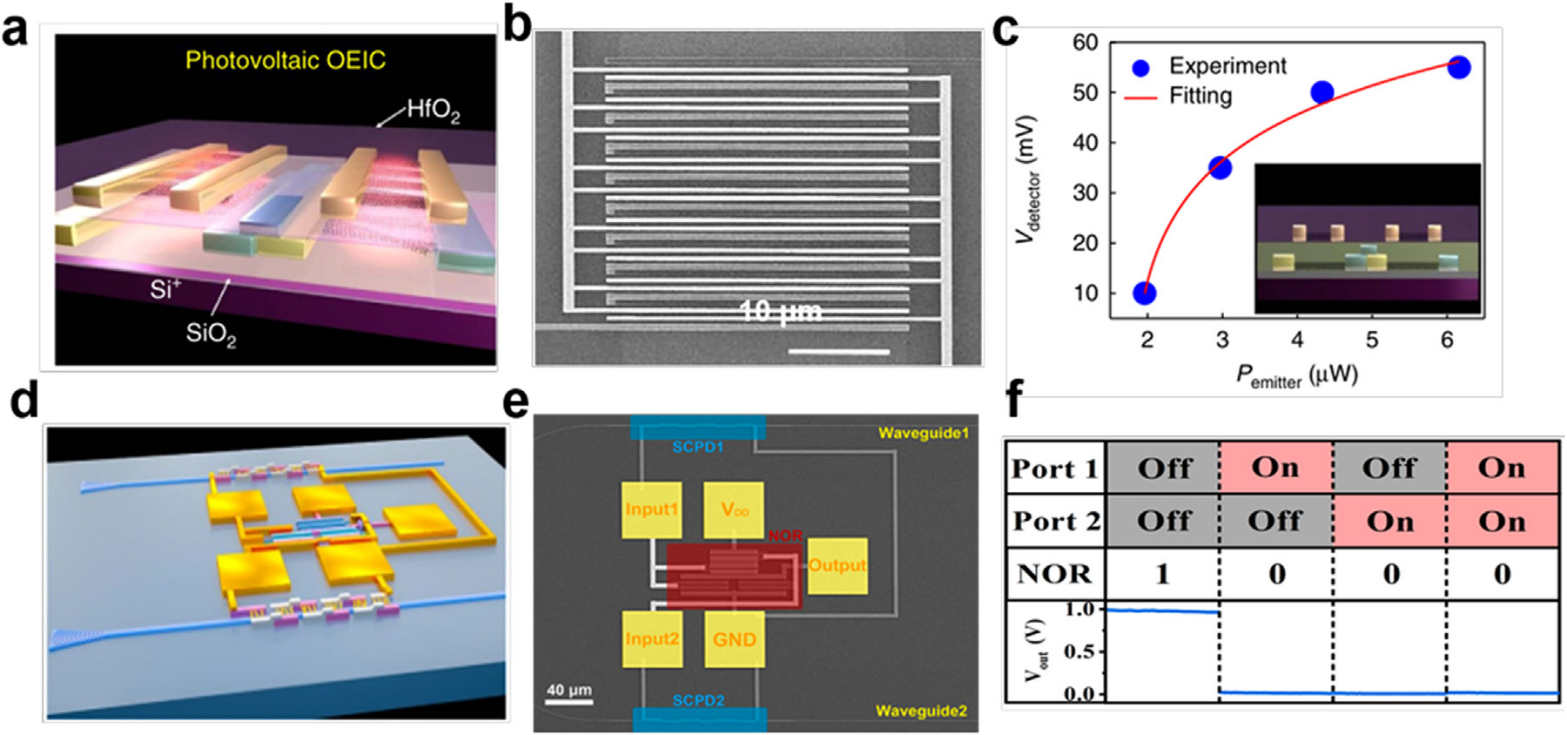
**CNT-based optoelectronic integrated systems.** (a) The schematic diagram OF CNT-based monolithic optoelectronic integration systems. (b) The SEM image of a vertical near-field optoelectronic integrated circuits. (c) Photovoltage of the bottom-layer detector versus the illumination power of the top-layer CNT emitter. Inset is the front-view graph of figure (a). Reproduced with permission from Ref [28], © *Springer Nature* 2017. (d) The schematic diagram of a silicon-waveguide-integrated CNT optoelectronic integrated system. (e) False color SEM image. (f) The true table and logic output of the system. Reproduced with permission from Ref [103], © *American Chemical Society* 2020.

Optical communication and optoelectronic integration. In the post-Moore era, on-chip electrically driven miniaturized optoelectronic devices and integrated circuits are pursued to construct compact communication systems, including high-speed light emitters, photodetectors, modulators, optical switches and electronic processing circuits. CNTs hold great potential in high-speed optical communications, due to the high carrier mobility and saturation velocity CNT, ultrasmall intrinsic capacitance, high absorption coefficient in the telecom band, picosecond intrinsic photoresponse time and doping-free CMOS compatible process [57,[136], [137], [138]]. In 2014, the first electrically driven ultrafast CNT light emitter based on blackbody radiation was reported with a response speed of 1–10 Gbps, more than 10^6^ times higher than that of conventional incandescent emitters and is comparable to that of light-emitting diodes or laser diodes [139]. This device also demonstrated 140 ps width pulsed light generation, experimental 1 Gbps and theoretical 10 Gbps modulation, and experimental 1 Mbps optical communication, opening new routes to higher integration densities in optical interconnects. As shown in Fig. 13a-c, Peng et al. realized the all CNT-based 3D optoelectronic integrated circuit, consisting of arrays of photovoltaic receivers, electrically driven transmitters and CMOS signal processing circuits [28,[140], [141], [142]]. These results show the potential of high-speed inter-layer data communication (10n^2^ Gbps for an *n* × n transmitters/receivers array). They later demonstrated the integration between silicon waveguides and a CNT optoelectronic system, as shown in Fig. 13d-e [103]. In this system, the waveguide-integrated CNT photodetectors exhibited 12.5 mA/W photoresponsivity at 1530 nm and they can be used to control the logic output of CNT logic inverters and NOR gates, enabling the CNT devices to be combined into a fiber-optic wavelength division multiplexing (WDM) system. In 2023, Wang et al. reported a CMOS-compatible ultrafast CNT photodetectors, demonstrating the 100 Gbit/s Nyquist-shaped on-off keying (OOK) signal transmission and a bandwidth over 60 GHz [29]. This work further paves the way for future CNT-based high-speed optical interconnects and optoelectronic-integrated circuits.

## 5 Summary and outlook

In this article, we summarizes the evolving device architectures of CNT photodetector over the past few years. From the initial scientific demonstrations of photocurrent in individual CNTs, various photodiodes and phototransistors towards high detectivity have emerged. These detectors based on different mechanisms mainly focus on improving EQE, and facilitating the separation and collection of photogenerated carriers. The CNT HGTFT that overcome the classic photodetection limits have been highlighted. This type of hybrid phototransistor allows both high absorption in the PbS QD/ZnO PN junction and efficient amplification of V_oc_ in the CNT TFT. At room temperature, the D* of 5.6 × 10^13^ cm Hz^−1/2^ W^−1^ (at 1300 nm wavelength and cutoff wavelength ∼1.5 µm) is comparable to that of the best existing uncooled InGaAs detectors. The HGTFT also holds great potential for fast response with a preliminary research result of 570 µs.

Besides, the accurate characterization of novel thin-film photodetectors is important for the progress of optoelectronic technology, which helps clarifying the physical principles guiding photodetector optimization, and makes a comparison of their performance more realistic. Precise measurements of the active radiation power and noise power spectra of photodetectors are required to effectively determine their detectivity. It is recommended that devices with exceptional performance should be sent to several different laboratories for the performance verification. Another possible approach to verify D* is to independently and directly determine the NEP. However, this approach may be difficult to implement in practice due to the requirements of ultra-low radiation power and ultra-low noise floor of the test system.

With unique optoelectronic properties, CNT photodetectors have been employed into several novel applications, including polarization-sensitive photodetection, optical synapses, optical communications and optoelectronic integration. However, focal plane arrays and camera prototype devices based on CNTs are still challenging. These rely on a high-performance CNT photodetector monolithically integrated with Si ROIC. Challenges include the realization of (1) uniform and high purity (e.g., 99.9999%) CNT films, (2) further increase of device sensitivity and speed, and (3) matched design of ROIC. With the above issues solved in the near future, CNT photodetectors may soon find their way toward real applications.

## Declaration of competing interest

The authors declare that they have no conflicts of interest in this work.
